# Fluorescent Submicron-Sized Poly(heptafluoro-*n*-butyl methacrylate) Particles with Long-Term Stability

**DOI:** 10.3390/molecules25092013

**Published:** 2020-04-25

**Authors:** Maciej Jarzębski, Przemysław Siejak, Monika Przeor, Jacek Gapiński, Anna Woźniak, Hanna Maria Baranowska, Jarosław Pawlicz, Elżbieta Baryła-Pankiewicz, Anna Szwajca

**Affiliations:** 1 Department of Physics and Biophysics, Faculty of Food Science and Nutrition, Poznan University of Life Sciences, Wojska Polskiego 38/42, 60-637 Poznań, Poland; maciej.jarzebski@up.poznan.pl (M.J.); przemyslaw.siejak@up.poznan.pl (P.S.); hanna.baranowska@up.poznan.pl (H.M.B.); 2 Department of Gastronomy Science and Functional Foods, Faculty of Food Science and Nutrition, Poznan University of Life Sciences, Wojska Polskiego 31, 60-624 Poznań, Poland; monika.przeor@up.poznan.pl; 3 Molecular Biophysics Division, Faculty of Physics, Adam Mickiewicz University in Poznań, Uniwersytetu Poznańskiego 2, 61-614 Poznań, Poland; gapinski@amu.edu.pl; 4 NanoBioMedical Centre, Adam Mickiewicz University in Poznań, Wszechnicy Piastowskiej 3, 61-614 Poznań, Poland; wozniaka@amu.edu.pl; 5 Department of Orthopedics and Traumatology, Poznan University of Medical Sciences, 28 Czerwca 1956 str. No. 135/147, 61-545 Poznań, Poland; jarekpawlicz@gmail.com; 6 Faculty of Health Sciences Pomeranian Medical University in Szczecin, Żołnierska 48, 71-210 Szczecin, Poland; elzpan@gmail.com; 7 Department of Chemistry, Adam Mickiewicz University in Poznań; Uniwersytetu Poznańskiego 8, 61-614 Poznań, Poland

**Keywords:** fluorinated particles, core–shell NPs, Rhodamine B, fluorescence, confocal microscopy

## Abstract

Fluorescent submicron particles of fluorinated methacrylate (HFMBA) with long-term stability have been synthesized and characterized with regard to their potential applications. Rhodamine B (RBITC) isothiocyanate was used as the fluorescent component. The core–shell structure of the particles effectively protected the dye against bleaching. HFBMA nanoparticle (NP) stability was confirmed after seven years of storage. Only slight differences were found in the polydispersity index (pdi) from 0.002 to 0.010. Particle size measurements were carried out using dynamic light scattering (DLS), nanoparticle tracking (NTA), and fluorescence correlation spectroscopy (FCS). The hydrodynamic diameter evaluated by different methods were in good agreement, respectively: 184–550 nm, 218–579 nm, and 236–508 nm. Particle and core morphology was estimated by using scanning and transmission electron microscopy (SEM and TEM). The ability to recognize particles in 3D as a reference sample in biological media has been confirmed by epifluorescence optical microscopy, confocal laser scanning microscopy, and super-resolution confocal microscopy (STED).

## 1. Introduction

Nanoparticles (NPs) were initially defined as structures with one or more dimensions in the range of 1–100 nm [1]. In practice, an increasing number of published works have extended the meaning of NPs into the submicron range up to 1000 nm. NPs with defined properties, i.e., size, shape, and biocompatibility nowadays have a higher impact on daily life [2,3,4,5]. One of the most interesting types of NP systems is the one based on a core–shell (CS) structure [6,7,8,9]. CS systems contain a core and shell/shells made from different materials [1,10,11,12,13]. NPs are efficient delivery systems compared to other carrier systems. The main advantage of NPs is their submicron size. Additionally, NPs can be easily manipulated in size and surface characteristics. They remain stable during long term storage and are able to incorporate both hydrophilic and hydrophobic molecules. Their high carrier capacity is combined with efficient carrier protection sustaining its activity [14]. On the other hand, there are various NP systems with the same basic material used in the whole particle; however, the layer-by-layer production—this process is also called “seed-growth” [15,16]—allows for the incorporation of different agents into chosen layers, e.g., fluorophores.

Fluorescent nanoparticles are widely used for fluorescence imaging in cells and tissues [17,18,19]. Using fluorescent NPs permanently eliminates unwanted sequestration, nonspecific binding by cellular biomacromolecules, and accidental accumulation of imaging agents. Experiments with stained LipImageTM 815 NP showed that the fluorescent factor accumulated mainly in the liver after 24 h, as well as in orthotopic tumors [20]. When using fluorescent CS nanoparticles in microscopic studies, great attention is paid to obtaining the right particle size and stability of the dye contained in them. One of the solutions is the preparation of fluorescent core–transparent shell particles [16,21]. In the case of polymerization during the seed-growth process, a small part of the fluorescent agent might be incorporated not only in the center of the particle, but also in the next core layer [22,23]. Incorporation of the dye in the typical radical polymerization reaction is difficult to control. For that reason, and to prevent the fluorescent agent from bleaching, a second polymerization reaction (“seed-growth process”) is usually applied. Authors [24] reported that spectroscopic characteristics of dye-doped silica nanoparticles indicate a much higher photostability and brightness in comparison to the nanoparticles with free uncoupled dyes. As an alternative to solid fluorescent NPs, alginate gel-based systems were proposed [25].

Fluorinated polymers and nanoparticles exhibit desirable properties, such as low refractive index [26], chemical and thermal stability, flame retardancy, low dielectric constant, and low frictional properties [27]. The free radical polymerization process was used for the synthesis of the new fluorinated polymers. Sabatini [28] proposed new fluorinated polymers exhibiting high UV photo and chemical resistance, which might be successfully used as coating materials for cultural heritage. Fluorinated nanomaterials are often considered for biomedical applications. They are proposed as a way to manufacture grafted surfaces for adhesion prevention of fungal pathogens, such as *Candida albicans* [29] which causes over 80% of candidiasis. Fluorinated composites with graphene have been used as possible insulators for flexible electronics [30]. Hybrid nanostructures based on fluorinated nanometer-sized silica particles have been proposed as a new material for self-cleaning, stain-resistant, nonfouling, abrasion-resistant coatings [31]. Molecules with 19F substituents are particularly attractive for use in drug tracking by 19F magnetic resonance imaging (MRI) because they produce no background signal in biological samples [32]. Therefore, nanoparticles containing fluorine atoms have the potential for imaging in drug delivery.

In this paper, we characterized fluorescent submicron-sized fluorinated methacrylate particles as particles exhibiting long-term stability. We focused on two main properties: fluorescent behaviour related to possible use for microscopic investigations, and the hydrodynamic size. Studies were performed using several techniques: dynamic light scattering (DLS), which was compared with fluorescence correlation spectroscopy (FCS), and nanoparticle tracking analysis (NTA). The particles were imaged by epifluorescence microscopy (EFM), confocal and high-resolution laser scanning microscopy (CLSM and STED), and electron microscopy (TEM and SEM). XPS, contact angle measurements (CA), steady-state fluorescence spectroscopy (SFS), and fluorescence lifetime measurements (FLT) were used as complementary methods. Furthermore, in this paper, we compared the results of hydrodynamic diameter values after synthesis and after a long period of storage. The fluorinated fluorescent polymer NPs from 1H,1H-heptafluoro-n-butyl methacrylate monomers with covalently bonded Rhodamine-B (RBITC) are useful and versatile systems for confocal laser scanning microscopy and fluorescence correlation spectroscopy. This has been confirmed by former studies of the structure and dynamics in colloidal systems [16].

## 2. Results and Discussion

### 2.1. Particle Size and Morphology Characterization

The synthesis of target fluorescent nanoparticles was carried out using the apparatus presented in the diagram in Figure S1 (Supplementary file 1) and a procedure based on literature data [16]. The resulting nanoparticles have a spherical shape, which is the result of the tendency of particles to form spherical micellar systems in the reaction mixture. In structures of this type, the hydrophilic heads are directed outwards, and the hydrophobic fragments inwards to the inner part of the NP structure. The crosslinking factor has a great influence on the stability of the obtained structures. In Table 1, we present the codes and descriptions of the NPs synthesized for this work.

After HBMA NPs preparation according to [16], we decided not to use any additional purification processes. After synthesis, HFBMA samples were only filtrated through the paper filter and stored in glass bottles. We noticed rapid particle agglomeration when contacted with a plastic pipette tip. Residues of surfactant (SDS) in the particle suspension might protect against aggregation and increase stability. During long-term storage, we did not observe aggregates in the tested NP solutions. Furthermore, the samples were mostly stored at room temperature and kept locked up and out of the sunlight. All measurements were carried out at very low concentration. Possible side effects should be negligible in such conditions [33].

The size of the HFBMA particle was determined using dynamic light scattering (DLS). As can be observed in Figure S2 (Supplementary file 1), the synthesized submicron particles showed high polydispersity, which increased when an additional polymerization process was performed for the core–shell structure preparation. It must be pointed out that samples showed stability after a few years of storage. We compared the results obtained directly after synthesis with those obtained after two months (Table 2).

Note that the hydrodynamic diameter values were determined using two different apparatus (ALV/CGS-3 with modified CONTIN software [34] and Zetasizer Nano-ZS90 with the Malvern software), which may affect the calculated hydrodynamic diameter values. Nevertheless, despite differences in mathematical software models, the results of the particle size measurements are consistent. The polydispersity effect of the core–shell samples were discussed in our previous paper [16]. In brief, during the second polymerization for core–shell structure preparation, an additional process of second nucleation occurred in some cases. For additional studies of our samples, nanoparticle tracking analysis (NTA) was applied. NTA allows measurement of the single-particle diffusion coefficient, which relates to hydrodynamic diameter in very dilute suspensions [35]. The weights of the size distributions measured by NTA and DLS are different, which was discussed in our former study [36]. Moreover, for such large particles, the size distribution obtained by DLS requires exact knowledge of the particle form factor, otherwise the result may depend on the scattering angle and the laser light wavelength [28].

**Figure 1:**
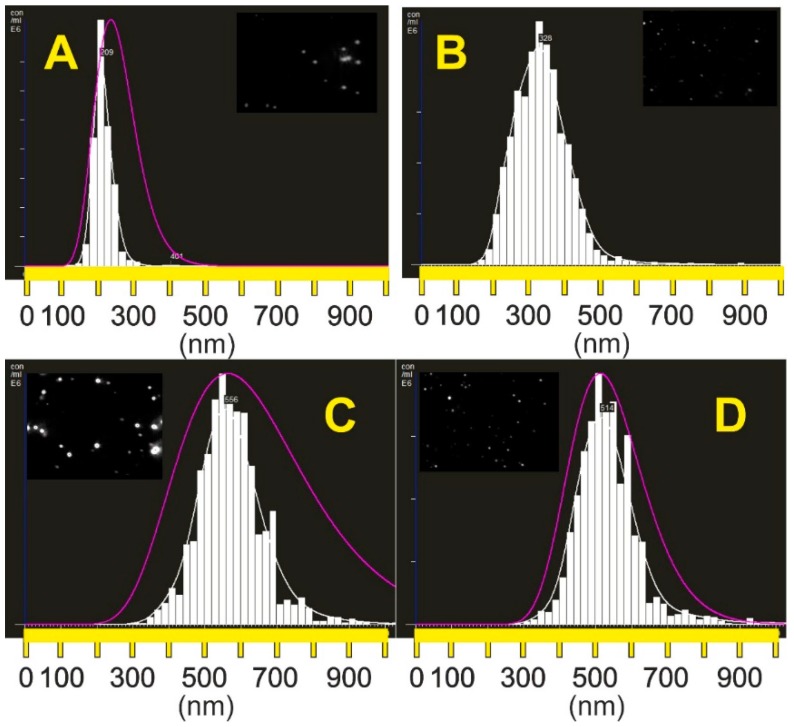
Particle size distribution of core H10 and H14 (**A**,**B**), core–shell H26 and H27 (**C**,**D**) particles obtained by NTA (pink curve—DLS Nanosight NS500 results; NTA video frames are shown in the inserts).

Particle size distributions evaluated by NTA are presented in Figure 1.

The results obtained during DLS, NTA, and FCS measurements are presented in Table 3. The FCS method is typically used for size determination of small (single nanometers) fluorescent particles, such as dyes or proteins [37,38]. In the case of particles comparable in size to the size of the confocal volume, diffusion times measured by FCS are not proportional to the particle size. A proper correction was proposed in a former study [39]. The proposed model was successfully validated for the polymeric and silica particles, whose diameters were larger than 500 nm [36]. In Table 3, we present the mean hydrodynamic diameters of the fluorescent core and core–shell particles determined by different techniques.

**Figure 2:**
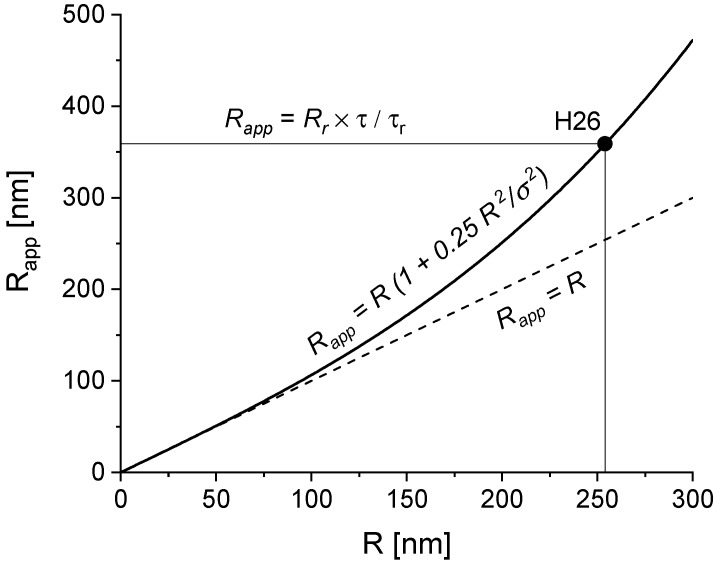
Illustration of the correction procedure of R_FCS_ for the H26 sample.

In Table 4, we list the parameters required to estimate the actual hydrodynamic radii of fluorescent particles based on the FCS diffusion times. The correction procedure is necessary when the actual particle radius is greater than the confocal volume size σ. To reduce the correction factor, an objective producing a large confocal volume (small numerical aperture) was used. The correction procedure is explained in Figure 2 for the data obtained for the H26 sample.

**Figure 3:**
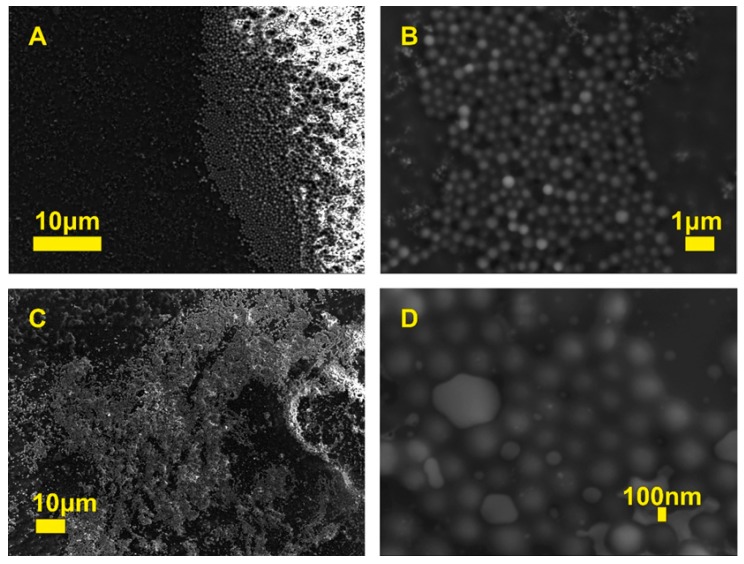
SEM images of HFBMA NPs at different magnifications: H26 (**A**,**B**), H27 (**C**,**D**).

The structure and morphology of the particles were evaluated by SEM and TEM microscopes. Figure 3 presents the HFBMA particles under different magnifications. The lighter areas of the samples might occur due to melting of the samples in the fragments most exposed to the electron beam, an effect sometimes observed in polymeric materials. Nevertheless, the synthesized submicron particles were spherical in shape. Visible in Figure 3D, a large divergence of particle size illustrates the relatively high polydispersity of the H27 sample. Note that SEM was applied only in the studies of whole particle morphology. The fluorescent cores alone were imaged using a TEM microscope.

**Figure 4:**
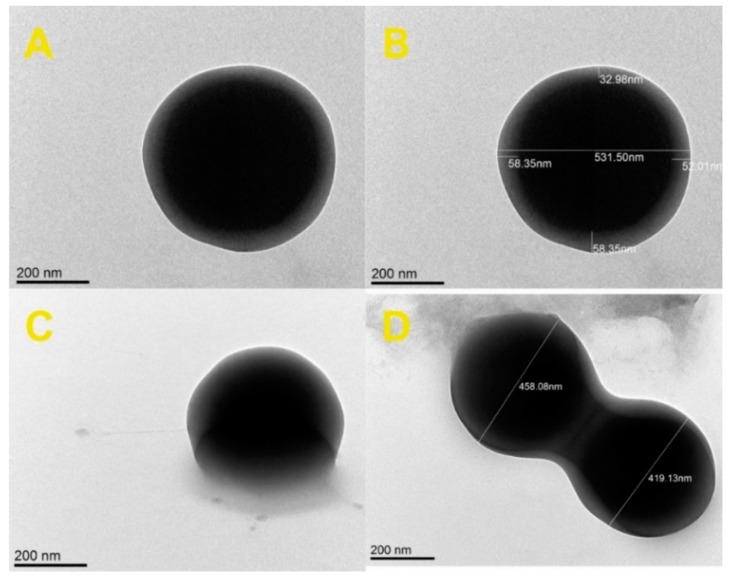
TEM images of HFBMA (H27) particles: single particle (**A**), the same single particle with dimensions (**B**), single particle image taken at +57° (**C**), particle twins (**D**).

Examples of such images are shown in Figure 4. The lighter parts cannot be a shell but differ z-slide (3D) of the particles. Using TEM is typical to recognize core–shell structures when the core and shell are made from different materials. Presence of remnant surfactant molecules can be observed in Figure 4C taken at the angle of 57°. Detailed studies of the morphology of individual particles showed the effect of second-phase particles: during the second polymerization process, twins of particles were created as a result of shell sharing between two cores. A similar effect was observed during the seed-growth process of submicron silica particles (see Supplementary Data 2 in [15]). Note that the detailed shape of the particles cannot be recovered from the studies based on diffusion measurements (DLS, NTA, FCS).

### 2.2. Spectroscopic and Contact Angle Analysis

#### X-ray Photoelectron Spectroscopy (XPS) and Contact Angle (CA) Analysis

**Figure 5:**
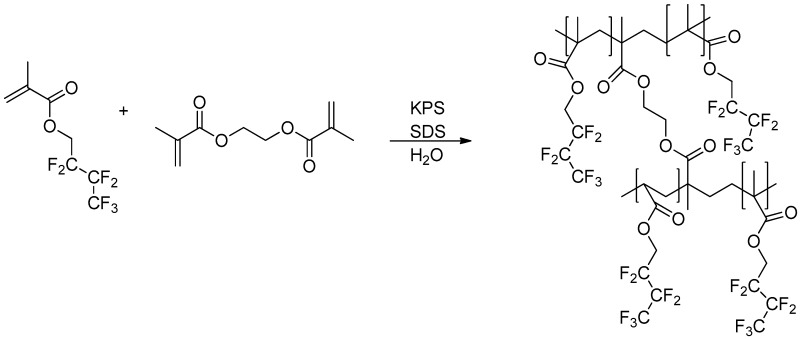
Schematic of HFBMA polymerization, in the presence of ethylene glycol-di-methacrylate (EGDMA).

The chemical composition of the NP films was confirmed by XPS measurements on partially covered glass slides. The NP films were fabricated as follows. First, the surface of the glass slides was cleaned and activated. Next, the NP films were formed by depositing drops of stock solution of appropriate NPs. The samples were allowed to dry on the glass slides in the N_2_ flow box. All of them have been tested both by XPS and CA analysis. In the present study, HFBMA core and core–shell NPs were prepared by radical polymerization in the presence of ethylene glycol-di-methacrylate (EGDMA) as a cross-linker (Figure 5).

**Figure 6:**
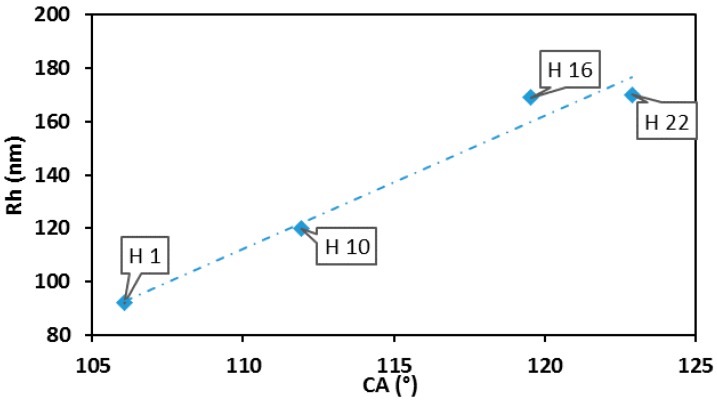
Static contact angles (°) HFBMA NP thin films versus R*_h_* (nm) of HFBMA NPs.

**Figure 7:**
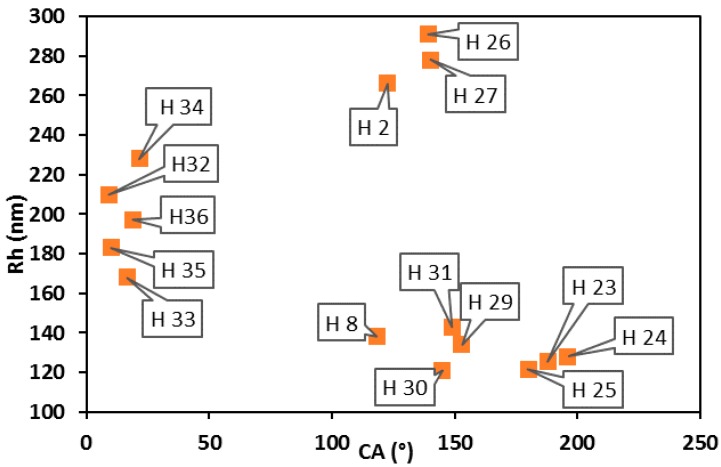
Static contact angles of HFBMA NP thin films correlated with *R_h_* values of NPs.

The content of monomer and cross-linker was evaluated by XPS and compared with the results of CA. The results of chemical composition measurements are summarized in Table 5. The results of the wettability tests are shown in Figure 6 and Figure 7.

The CA values of Core NP films increased from 106° to 123°, while the hydrodynamic radius *R_h_* increased from 92 nm to 170 nm. The correlation is linear, which indicates that as the radius of core NPs increases, the hydrophobicity of the fluorinated NPs film increases, too. The dependence of CA on *R_h_* for core–shell NPs does not show similar behavior (Figure 7). All core–shell NP thin films exhibited hydrophobic properties, with water contact angles higher than 100°, except NP SAM of H34 and H36 (22° and 19°).

The NPs with *R_h_* over 200 nm formed a glossy thin film of nanoparticles stuck together with hydrophilic and hydrophobic characteristics. Some of them retained water inside the polymer units, while others expelled the water. The films of particles with *R_h_* < 200 nm had a powdery form.

**Figure 8:**
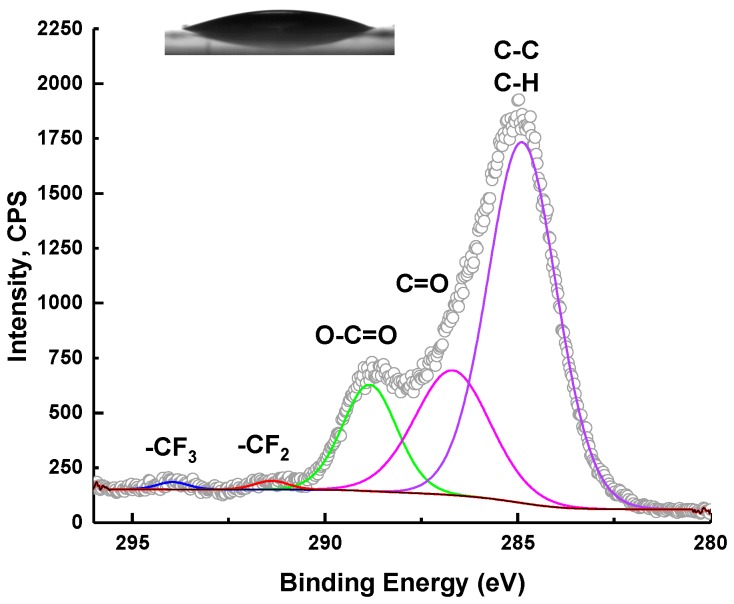
C 1s XPS spectra and static water contact angle (19°) of thin H36 NP film on modified glass slides.

**Figure 9:**
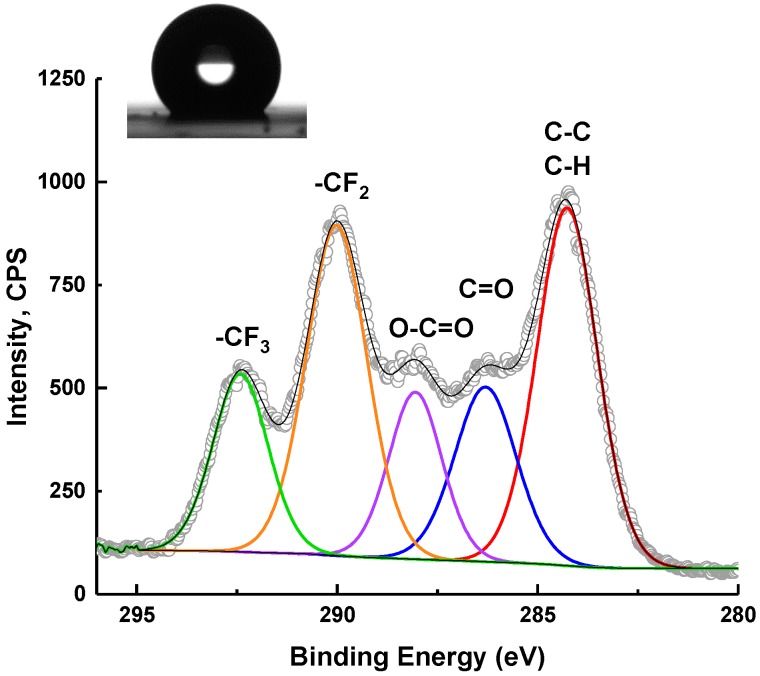
C 1s XPS spectra with static water contact angle (118°) of H8 NP films on modified glass slides.

It seems that the surface wettability of HFBMA NPs can be easily manipulated (Figure 8 and Figure 9).

### 2.3. Fluorescent Behavior Investigations

#### Fluorescent Behavior Investigations

Rhodamine B isothiocyanate is one of the most frequently used fluorescent dyes, even though it is not the best dye in terms of the small Stokes shift [40]. Various types of Rhodamine (B, 6G, 101 etc.) are used as a tool for designing a number of model systems for microscopic investigations. For example, Guldbrand et al. [41], based on two-photon fluorescence microscopic studies of rhodamine and RBITC, has proposed a model of fluorescent molecule diffusion in the skin. Rhodamine 6G has been used as a reference sample for particle size determination by the FCS technique in previous studies [21]. The fluorescence quantum yield of xanthene dyes, especially rhodamines, and its derivatives is from 0.36 to 0.98 depending on the exact structure and solvent [42]. The modification of Rhodamine B by an isothiocyanate group brings opportunities to easily label various types of proteins [43], polysaccharides [44], and nanoparticles [45,46].

**Figure 10:**
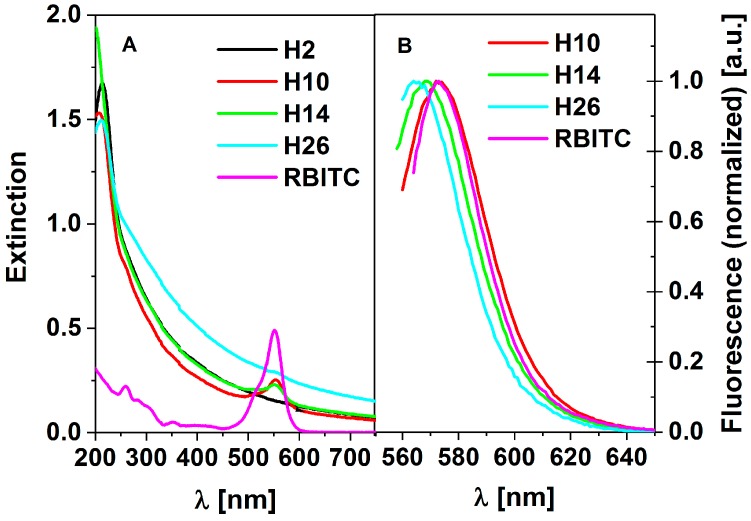
Extinction (**A**) and normalized fluorescence (**B**) spectra of H2, H10, H14, H26, and RBITC samples; no fluorescence for H2 was observed (**B**).

The goal of the present study was to obtain fluorescent particles with high dye stability during microscopic investigations preserved over long storage periods. The presence of rhodamine-B-isothiocyanate (RBITC) in each of the investigated systems was confirmed by UV-VIS spectroscopy and fluorescence emission spectra measurements. Recorded UV-VIS spectra were characteristic for samples containing large, strongly scattering particles, which we assigned to our HFBMA particles. For each examined system containing RBITC, an additional peak at wavelength 550–560 nm appeared, assigned to the dye. Moreover, clear fluorescence spectra characteristic of RBITC were recorded. Additionally, fluorescence excitation spectra were collected in order to distinguish dye spectra from the scattering signal (not shown). The recorded extinction and fluorescence emission spectra of the investigated systems are presented in Figure 10.

As can be seen from Figure 10A, the background shape of the spectra arises from HFBMA’s scattering properties. The extinction spectrum of the polymer alone is given in Figure 10A as a black line. No fluorescence for H2 samples (bulk HFBMA) was detected. Particles with no clear fluorescent signal were recorded for sample H26 due to an additional second polymerization process. Sample H26 was prepared during the “seed-growth” process.

Neither the amount of RBITC embedded in the nanoparticles, nor the location of dye could be precisely defined.

**Figure 11:**
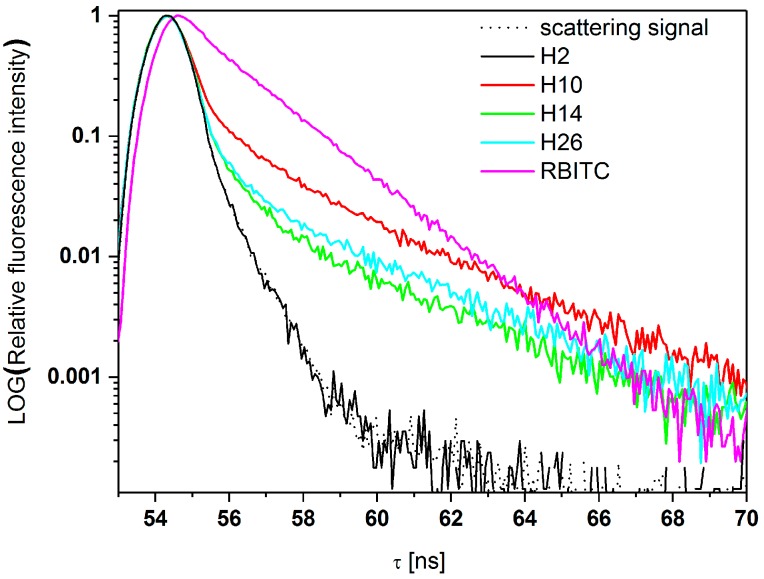
Fluorescence time decays of investigated samples.

From Figure 11, it follows that encapsulation of RBITC in HFBMA leads to a shift of the peak position to lower wavelengths. This suggests a strong influence of the shell surface on RBITC’s fluorescence properties. However, it should be stressed that the alteration is within the range of 10 nm. The alteration of fluorescence band location is not crucial for most applications, including medical procedures, considering the abilities of detectors. The exact wavelengths of the shifted fluorescence peaks are shown in Table 6.

The recorded kinetics of fluorescence decay (semilogarithmic scale) are shown in Figure 11. The fluorescence decay curves (semilogarithmic scale) are shown in Figure 11.

From Figure 11, it follows that HFBMA polymer NPs do not show fluorescence, but only scatter light similarly to a scattering standard. The scattering process is also manifested by the first curved region of the time decay curve for HFBMA+RBITC samples. Due to negligible scattering efficiency, the curved shape is hardly noticeable for the RBITC solution itself. The kinetics are linear in the whole detection range.

As can be seen from Figure 11 and Table 6, the influence of the thick HFBMA layer surrounding the fluorescent probe on fluorescent properties is significant. The change of a fluorescent NP’s lifetime can be considered as an advantage for some applications, due to the facilitation of fluorescence detection. It seems possible to easily modify the fluorescence lifetimes of RBITC according to individual requirements, by modifying the synthesis time or simple shell coverage engineering of NPs. Incorporation of RBITC inside the optically transparent fluorinated particles allowed detailed studies of the NPs using NTA in scattering and fluorescence mode (Figure S3 Supplementary file 1).

**Figure 12:**
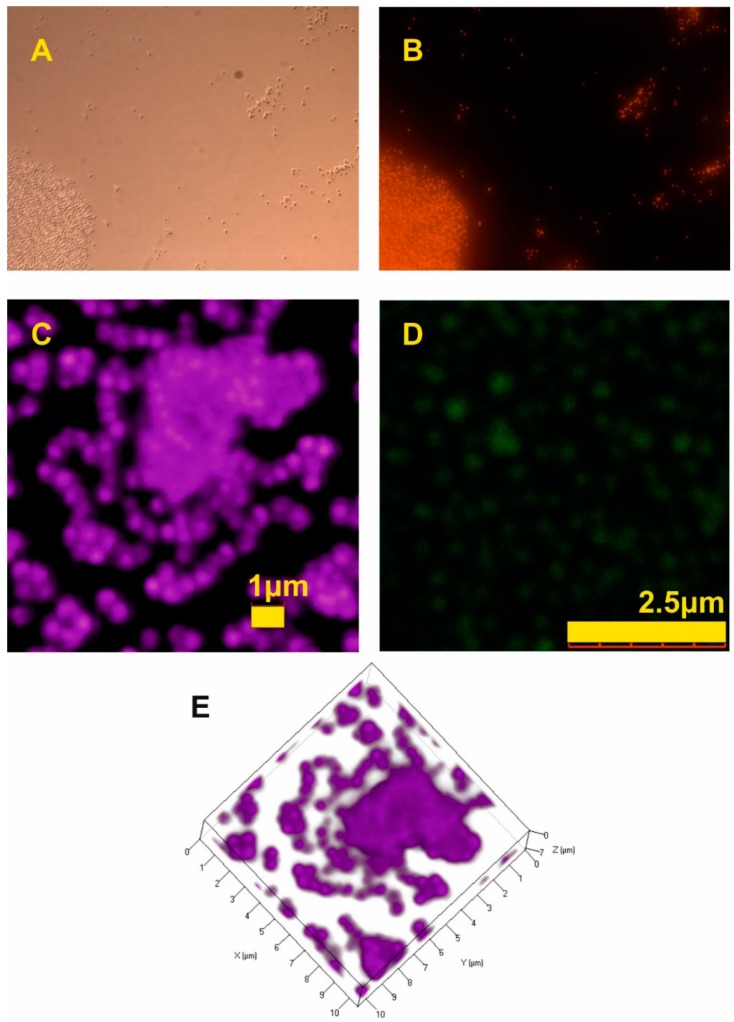
Microscopic images of HFBMA NPs: bright field microscopy (**A**), epifluorescence microscopy (**B**), confocal laser scanning microscopy (**C**), STED (**D**), a 3D picture from CLSM (**E**).

The most important results were obtained using different microscope fluorescent techniques. The epifluorescence image (Figure 12B) was compared to the regular bright field image (Figure 12A). Most of the studied NPs were large enough to be distinguished even by these two simple microscopy techniques, which are often used for preliminary tests, i.e., in cell cultures. For detailed studies, confocal laser scanning microscope was applied. In a previous study [14], we performed basic tests for core–shell structure validation. With CLSM, only the range of the size can be estimated. For better accuracy, a super-resolution STED microscope (Figure 12D) is recommended. Due to longer exposure times in STED imaging, the fluorescent particles should pose high fluorescence stability. Figure 12E shows a 3D picture of sedimented NPs obtained by CLSM. The possibility of the recognition of the particles in 3D is extremely important for possible biomedical application, i.e., to verify when particles fall inside the cells or concentrate at the surface or in the selected organelle.

## 3. Materials and Methods

### 3.1. Materials

Rhodamine-B-isothiocyanate (RBITC), allylamine 99.5%, sodium dodecyl sulfate (SDS) 99%, and methanol (99.8%) were purchased from Sigma–Aldrich and used without further purification. 1H,1H-Heptafluoro-*n*-butyl methacrylate (HFBMA) 97% was purchased from SynQuest Labs (Alachua, FL, USA) and crosslinker ethylene glycol-di-methacrylate (EGDMA) 98% was purchased from Acros Organics (Geel, Belgium).

### 3.2. Sample Preparation

Nonfluorescent samples were synthesized using the method proposed in [47,48], The synthesis procedure described in detail in [14] was applied for the preparation of fluorescent nanoparticles. A series of core and core–shell submicron particles were made. The authors decided to keep their original code names in this article for easy comparison in further studies. The representative samples in this paper are described in Table 1 (additional comments and details are pointed directly in the text in case of using other samples). The model of the prepared samples is presented in Figure S1 (Supplementary file 1).

The resulting precipitate was removed by filtration, and the filtrate was stored in glass jars. A reference sample of Rhodamine-B-isothiocyanate (RBITC) was prepared as follows: 42.9 mg of RBITC was diluted in methanol. As-prepared RBITC stock solution was used for further investigations. The stock samples of NPs were diluted with double distilled water to obtain transparent solutions suitable for the fluorescence techniques. The fabrication of NP films requires clean and hydroxylated glass coverslips (RVFM Company, Colchester, UK). The 2 cm × 2 cm glass wafers were treated with piranha solution (H_2_SO_4_:H_2_O_2_ 1:1, 15 min at 85 °C), followed by extensive rinsing with deionized water, before the film deposition.

### 3.3. Methods

#### 3.3.1. Hydrodynamic Diameter and Zeta Potential Determination

The hydrodynamic radius of HFBMA particles was determined by dynamic light scattering (DLS) directly after the synthesis and for selected samples also after a couple of months using a DLS setup (ALV-Laser vertriebsgesellschaft, Langen, Germany), based on a ALV/CGS-3 goniometer (Langen, Germany), a Helium–Neon laser (632.8 nm) and ALV 6000 series correlator (Langen, Germany). All measurements were performed at 20 ± 0.1 °C. The light scattering autocorrelation functions were obtained for scattering angles between 20° and 150° with an angular step of 5°. The diffusion coefficients were obtained directly from the autocorrelation functions by using CONTIN algorithm for multiexponential decay analysis [25] built into the ALV-Correlator software (version 3.0).

For a detailed study of particle size distribution, nanoparticle tracking analysis was applied. The measurements were performed using NanoSight NS500 analyzer (Malvern Panalytical, Malvern, UK) equipped with a 405 nm laser. The recording time of moving particles was ranged from 30 to 215 s. The results were obtained using the fluorescence mode. Only particle tracks longer than 20 movie frames were taken for the diffusion analysis.

Fluorescence correlation spectroscopy (FCS) measurements were recorded by a Zeiss LSM 780 + ConfoCor3 with Olympus LD 40×/0.60 objective, excitation at 514 nm, emission at 530–600 nm. Samples were measured in Nunc™ Lab-Tek™ Chambered Coverglass systems (Thermo Scientific™) at 25 °C. The obtained correlation functions were analyzed using a standard two-component model. As a reference sample, RBITC water solution without adjusted concentration was used (for more details see in result and discussion section). Hydrodynamic radius in DLS, NTA, and FCS measurements was found indirectly from the diffusion coefficient using the Stokes–Einstein equation [49]:

$$D=\;\frac{k_{B}T}{6\pi \eta R_{h}}$$

where *R_h_* is a hydrodynamic radius, *k_B_* is the Boltzmann constant, *η* is the solvent viscosity, and *T* is temperature.

“Fresh measurements”, which means validation of selected samples’ stability, were performed with Zetasizer Nano-ZS90 (Malvern Panalytical, Malvern, UK). Hydrodynamic diameters (d_H_), as well as zeta potential (ζ) values of prepared particles, were measured. The DLS autocorrelation functions were registered from the scattered light recorded at an angle of 90°. Prior to the measurements, the samples were equilibrated at 25 °C for 5 min in the measurement cell. The average values from 10 measurements and particle size distribution were taken into the analysis and further presented.

Zetasizer 2000 from Malvern Instruments was applied for zeta potentials determination. A few droplets of highly diluted samples were immersed in 0.002 M TRIS solution.

#### 3.3.2. XPS

The chemical composition of the thin films of H8, H16, H27, and H36 on glass slides was examined using a SPECS (Berlin, Germany) spectrometer equipped with a Al K-α source emitting photons of energy of 1486.74 eV and a hemispherical analyzer (PHOIBOS 150) set to the pass energy of 20 eV. The XPS system base pressure was 1–4 × 10^−8^ Pa. The internal calibration of the XPS spectra was done due to C–C binding energy value of 284.6 eV. Chemical composition was resolved by a curve fitting (CASA XPS^®^ software) procedure using a sum of Gaussian (70%) and Lorentzian (30%) functions. The secondary electron background was subtracted via the Shirley procedure.

#### 3.3.3. Contact Angle

The wetting angle study was undertaken in order to establish the hydrophobicity of a thin film of HFBMA particles. The NP films were formed by depositing consecutive drops (10 μL) of water solution on the clean, oxidized glass substrate and allowing the solvent to evaporate. The surface drop contact angles of the covered glass surfaces were measured using an OCA 15+ Contact Angle Measurement System (DataPhysics Instruments GmbH, Filderstadt, Germany). Measurements were performed at different sites on each surface. A drop (0.2 µL) of water was deposited using a microsyringe. The final values were averages of at least five CA measurements on the same sample. The angles were determined using the Laplace–Young equation approximately 30 s after deposition of the water drop.

#### 3.3.4. Microscopic Investigations

For shape and morphology studies, scanning electron microscopy (SEM, Jeol, Tokyo, Japan, model 7001TTLS) was used. The maximum applied acceleration voltage was 30 kV and a maximum resolution of 1.5 nm was achieved. For detailed investigations, transmission electron microscopy (TEM Jeol, 1400, Tokyo, Japan) 1400 with maximum accelerating voltage 120 kV and maximum resolution 0.2 nm) was used.

The fluorescent behavior of the samples was investigated using light microscopy (LM). An Axio Scope.A1(Zeiss, Oberkochen, Germany) in transmission and fluorescence mode was used. A confocal laser scanning microscope (CLSM) Zeiss LSM 780 NLO (Oberkochen, Germany) and Leica TCS SP5 (Wetzlar, Germany) confocal fluorescence microscopy system equipped with high-resolution (stimulated emission depletion STED) mode were also used.

#### 3.3.5. Fluorescence and UV-VIS Spectroscopy

Fluorescence emission and excitation spectra were obtained using a Shimadzu RF 5001PC fluorometer (Kyoto, Japan). All the spectroscopic measurements were performed at ambient conditions, in a 1 cm × 1 cm quartz cuvette (HELMA). Samples were excited at UV and VIS region (correlated with the absorption peak of polymer and RBITC, respectively) and emission was measured over the range up to 700 nm with 3 nm excitation and emission slits. A 90° (L-shaped) geometry excitation to emission beam was used. Extinction spectra were recorded with the use of Shimadzu UV-1201 (Kyoto, Japan) UV-VIS spectrometer.

#### 3.3.6. Fluorescence Lifetime

Fluorescence lifetime measurements were carried out with a PicoQuant TimeHarp 100 PC-board for time-correlated single photon counting with 72 ps/channel resolution. The laser diode PLS -8-2-826 was used as an excitation source. The maximum of diode emission is centered at 575 nm and pulse full width at half maximum (FWHM) is 1.064 ns. The LED was powered by a PDL 800-D driver; the emission was detected by a PMA182 photosensor head (all the instruments were from PicoQuant, (Berlin, Germany). Data analysis was carried out by an exponential deconvolution method using a nonlinear least-square fitting program. The goodness of fit was estimated using χ^2^ values.

## 4. Conclusions

Our studies confirmed the long-term stability of the fluorescent fluorinated submicron particles. The results showed that the proposed fluorescent dye, Rhodamine B, can be well protected from bleaching using the fluorinated polymeric matrix. The presented results showed that for detailed studies of NP behavior in various conditions, more than one particle size determination method should be applied. The values of hydrodynamic diameter evaluated by different methods, such as DLS, NTA, and FCS were in good agreement. SEM was applied in the studies of particle structure and morphology. The synthesized submicron particles were spherical in shape. The shape of fluorescent cores was imaged using a TEM microscope. Detailed studies of the morphology of individual particles showed the effect of second-phase particles. The protective properties of the fluorinated shell allow studying the synthesized fluorescent particles by STED and confocal microscopy imaging. High dye and size stability of the synthesized fluorescent fluorinated particles allows confocal and STED microscopy investigations.

## Figures and Tables

**Table 1:** Description of the nanoparticle (NP) samples.

Sample	Description
H1	Transparent core
H2 ibH8	Transparent core with a transparent shell ibFluorescent core with a transparent shell
H10, H14, H16, H22	Fluorescent core
H26, H27, H36	Fluorescent core with a transparent shell
RBITC	Rhodamine-B-isothiocyanate

**Table 2:** Fluorinated methacrylate (HFMBA) samples studied by dynamic light scattering (DLS) after different sample exposure time.

Sample	Time						
	As-Prepared Samples *	Six Years of Storage **			Seven Years of Storage **		
	Average Diameter d (nm)	z-ave (nm)	pdi	Mean Peak (nm)	z-ave (nm)	pdi	Mean Peak (nm)
H1	184	219	0.022	228	223	0.014	232
H2	532	305	0.037	316	302	0.039	315
H10	188	248	0.033	260	250	0.022	261
H14	326	348	0.041	362	366	0.036	385
H26	550	592	0.054	618	570	0.046	586

* ALV/CGS-3 (ALV, Langen, Germany); ** Zetasizer Nano-ZS90 (Malvern Panalytical, Malvern, UK)

**Table 3:** Hydrodynamic diameters, FCS, and zeta potential (ξ) measured by DLS and NTA (fluorescence mode).

Sample	Hydrodynamic Diameter (nm)				
	DLS	NTA (mean)	NTA (mode)	FCS	ξ (mV)
H1	184	---	---	---	−61.7
H2	532	---	---	---	−71.6
H10	188	218	209	236	−71.2
H14	326	338	328	306	−63.8
H26	550	579	556	508	−67.8
H27	540	539	514	490	−72.3

**Table 4:** FCS correlation times τ_diff_, apparent hydrodynamic radii *R_app_*, corrected hydrodynamic radii *R_corr_* and the values of the correction factor.

Sample	τ_diff_ [ms]	*R_app_ = R_r_* × τ/τ_r_	*R_corr_*	Correction Factor
RBITC	0.0911	0.568	0.568	1.00
H10	20.14	126	116	1.08
H14	28.16	175	153	1.15
H26	57.64	359	254	1.41
H27	54.44	339	245	1.38

**Table 5:** O 1s, and C 1s core-level signals acquired from a H 16 and H 27 sample.

Sample	C 1s					O 1s	
	C–H	C–O	C=O	C–CF_2_	C–CF_3_	C–O	C=O
H16	284.4 eV	+1.9	+4.3	+8.9	+11.5	531.0 eV	+1.2
H27	284.4 eV	+1.0	+4.4	+8.7	+11.7	531.3 eV	+1.0

**Table 6:** Fluorescence excitation and emission wavelengths (λ) and shift of fluorescence band for the H10, H14, and H26 NPs.

Sample	λ (nm)		Fluorescence Band Shift (nm)
	Excitation	Emission	
RBITC	559	573	14
H10	555	573	18
H14	555	569	14
H26	555	565	10

## Supplementary Materials

The supplementary materials are available online (Supplementary file 1).
