# Supplementary material for: Fluorescent Submicron-Sized Poly(heptafluoro-n-butyl methacrylate) Particles with Long-Term Stability
Source: Molecules. 2020 Apr 25;25(9):2013. doi: 10.3390/molecules25092013 (PMC7249074; doi:10.3390/molecules25092013)
Supplement: Supplementary file 1 [file molecules-25-02013-s001.pdf]

Supplementary file 1

Fluorescent Submicron-Sized Poly(heptafluoro-*n*-butyl methacrylate) Particles with Long-Term Stability

Maciej Jarzębski [1], Przemysław Siejak [1], Monika Przeor [2], Jacek Gapiński [3], Anna Woźniak [4], Hanna Maria Baranowska [1], Jarosław Pawlicz [5], Elżbieta Baryła-Pankiewicz [6] and Anna Szwajca [7,*]

1 Department of Physics and Biophysics, Faculty of Food Science and Nutrition, Poznan University of Life Sciences, Wojska Polskiego 38/42, 60-637 Poznań, Poland; maciej.jarzebski@up.poznan.pl (M.J.); przemyslaw.siejak@up.poznan.pl (P.S.); hanna.baranowska@up.poznan.pl (H.M.B.)
2 Department of Gastronomy Science and Functional Foods, Faculty of Food Science and Nutrition, Poznan University of Life Sciences, Wojska Polskiego 31, 60-624 Poznań, Poland; monika.przeor@up.poznan.pl
3 Molecular Biophysics Division, Faculty of Physics, Adam Mickiewicz University in Poznań, Uniwersytetu Poznańskiego 2, 61-614 Poznań, Poland; gapinski@amu.edu.pl
4 NanoBioMedical Centre, Adam Mickiewicz University in Poznań, Wszechnicy Piastowskiej 3, 61-614 Poznań, Poland; wozniaka@amu.edu.pl
5 Department of Orthopedics and Traumatology, Poznan University of Medical Sciences, 28 Czerwca 1956 str. No. 135/147, 61-545 Poznań, Poland; jarekpawlicz@gmail.com
6 Faculty of Health Sciences Pomeranian Medical University in Szczecin, Żołnierska 48, 71-210 Szczecin, Poland; elzpan@gmail.com
7 Department of Chemistry, Adam Mickiewicz University in Poznań; Uniwersytetu Poznańskiego 8, 61-614 Poznań, Poland; anna.szwajca@amu.edu.pl
* Correspondence: anna.szwajca@amu.edu.pl; Tel.: +48-61-829-1820

Chemistry Methods

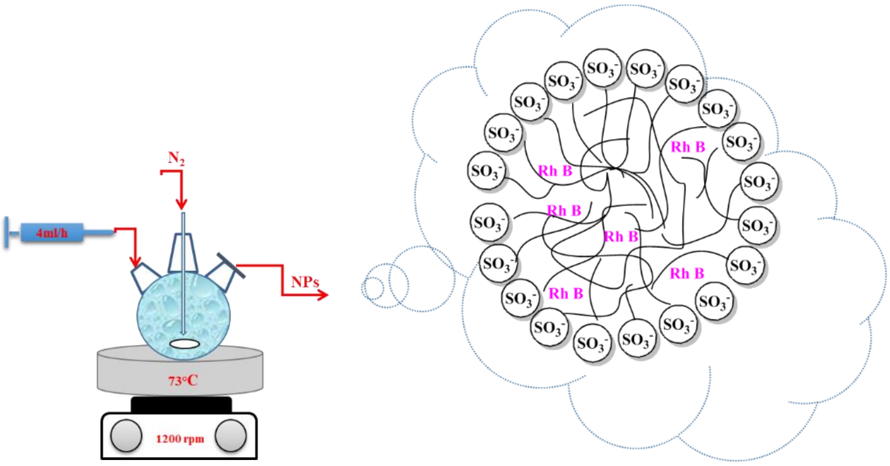

Figure S1. Model of polymer nanoparticles structure and reaction system scheme.

Particles size distribution by DLS

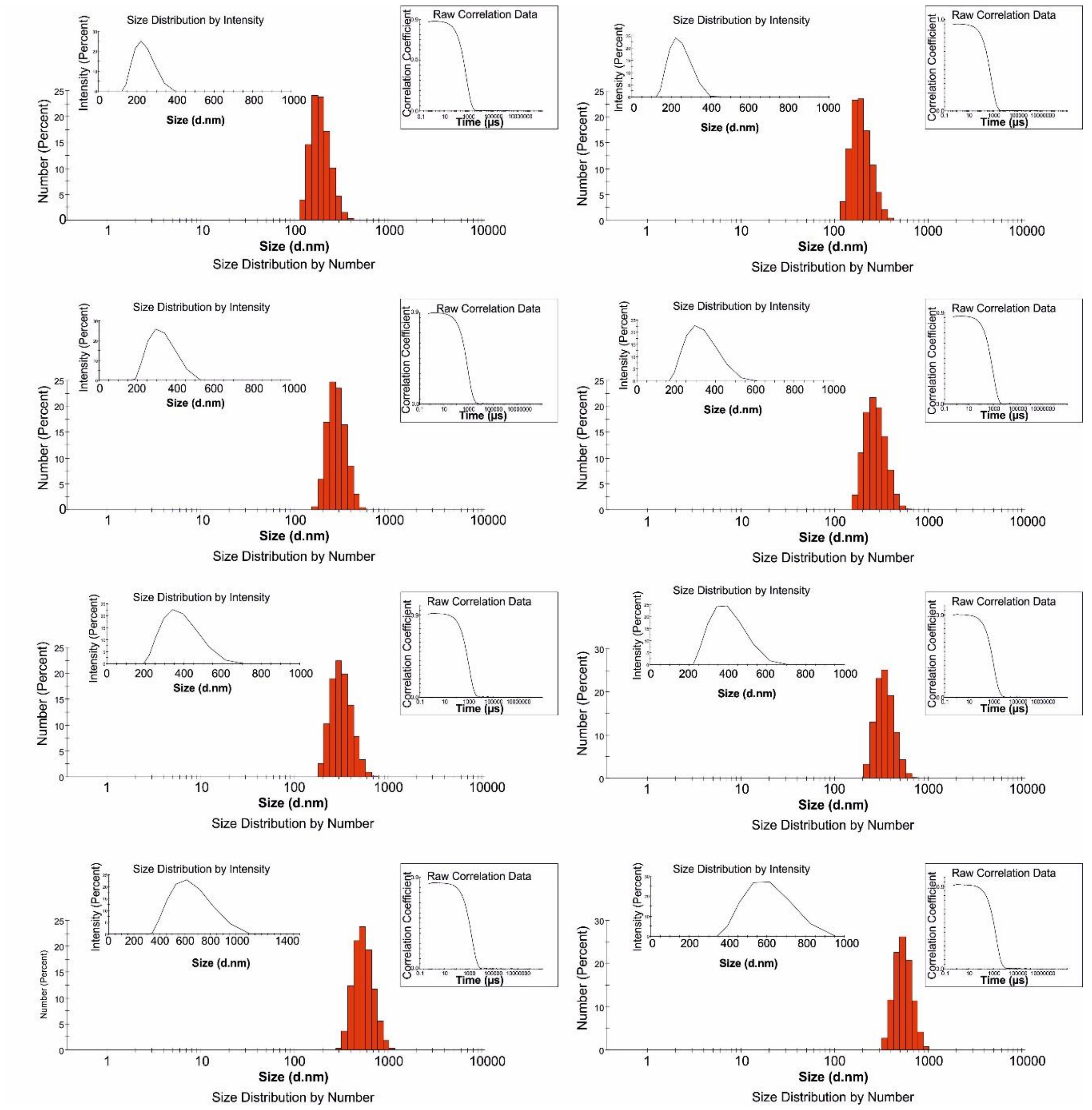

Figure S2. Particles size distribution by DLS (left side – sample after 1 year exposure time, right side – as-prepared samples) from top to down H1, H2, H14, H26 respectively.

Nanoparticle tracking analysis (NTA)

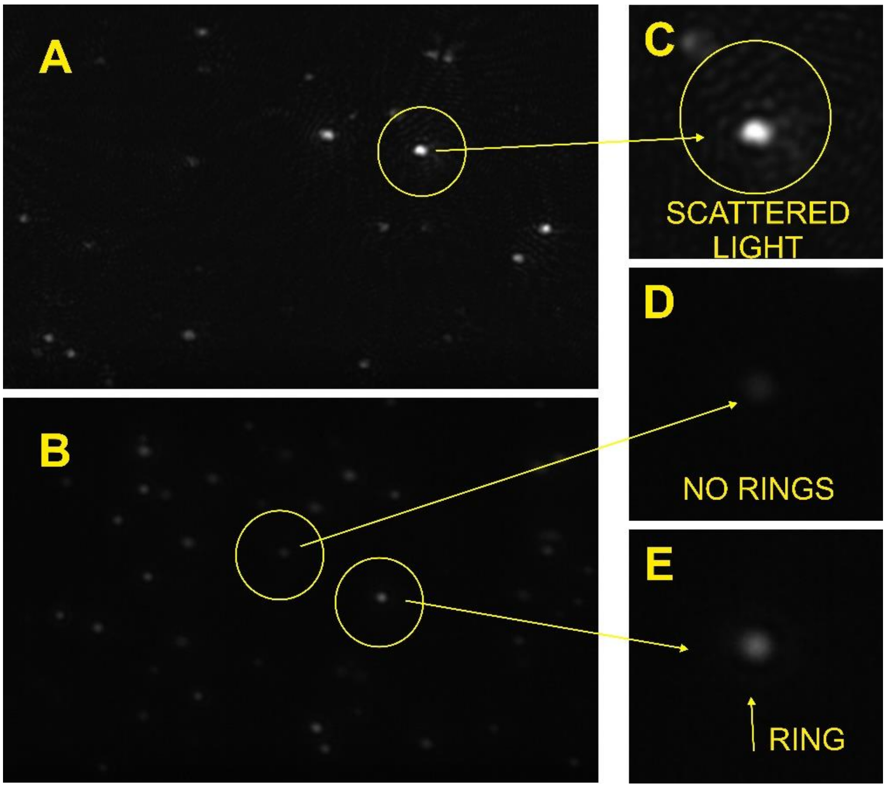

Figure S3. Sample frame obtained during NTA measurements A. scattering mode, B. fluorescence mode, C. large particle with scattered light signals, D. "ideal particle" for NTA. E. particle with scattered light ring.

Incorporation of RBITC inside the optically transparent fluorinated particles gave an opportunity for detailed studies of the NPs using NTA in scattering (Fig. S3A) and fluorescence mode (Fig. S3B) - for more details see p.2.1. Here should be pointed out that RBITC dye is quite easy to excitation. For the measurements, the 405nm laser wavelength was applied and strong scattered light signals from the particles were registered (Fig. S3B). Figure 14 showed possible 3 the most possibilities of particle scattering light signals (base on H26 sample): Fig. S3D – "ideal particle" (here registered in fluorescence mode) without any rings (Fig. S3E) and additional scattered light signals (Fig. S3C). Note that larger particles scatter more light and possible artefacts might occur during NTA analysis (Fig. S3A and S3C). We noticed that depending on the software (in our experiments NanoSight NTA2.3), sometimes scattered light might be interpreted as a very small particle. This case is typical for highly polydispersed samples (as presented here). If the signals from particles are registered in fluorescence mode (using dedicated wavelength and filters), the impact of the scattered light for the particle size distribution is strongly decreased.
